# Preventing central venous catheter-related bloodstream infections through implementation of a bundle intervention in the developing world: a quasi-experimental study

**DOI:** 10.1186/2047-2994-4-S1-O24

**Published:** 2015-06-16

**Authors:** MG Menegueti, AM Laus, M Auxiliadora-Martins, GG Gaspar, KMM Ardison, OA Martins-Filho, A Basile-Filho, F Bellissimo-Rodrigues

**Affiliations:** 1 Hospital das Clínicas da Faculdade de Medicina de Ribeirão Preto da Universidade de São Paulo (HCFMRP/USP), São Paulo, Brazil; 2 Escola de Enfermagem de Ribeirão Preto USP, São Paulo, Brazil; 3 HCFMRP/USP, São Paulo, Brazil; 4 FUNCAPE, Brazil; 5 Fundação Oswaldo Cruz, Rio de Janeiro, Brazil

## Introduction

Central venous catheter-associated bloodstream infections (CVCBSI) are among the most frequent health-care associated infections worldwide.

## Objectives

To investigate the effectiveness of a bundle intervention on reducing the rates of CVCBSI in critically ill patients admitted in a tertiary care hospital of Brazil.

## Methods

Quasi-experimental study designed to evaluate the impact on CVCBSI rates of implementing the following measures: training program that aimed to correct practices related to CVC insertion, manipulation, and maintenance, following the guidelines of the Centers for Disease Control and Prevention (CDC). Applying a checklist for each CVC insertion, bearing in mind a) the use of maximal sterile barriers during insertion, b) appropriate hand hygiene, c) insertion site disinfection and antisepsis, d) choice of catheter insertion site and reassessing the need for the central access on a daily basis.

## Results

We examined 123 checklists before and 155 checklists after implementation of the training program. Prior to the training, 71% of the staff members disinfected their hands using chlorhexidine 2% handwashing, 11% used alcohol hand rub based on ethanol 70%, 7% used a combination of both, and 7% performed an incorrect technique; hand hygiene was not verified for 4%. Following the training, 75, 9, and 14% of the staff used chlorhexidine, alcoholic solution, and a combination of chlorhexidine and alcoholic solution for hand disinfection, respectively; hand hygiene was not verified for 2% of the staff members. CVCBSI incidence was 9.3 and 5.1 per 1,000 catheter-days before and after the training program, corresponding to a reduction in CVCBSI by 4.3 episodes per 1,000 catheters-day (CI 95%: 2.27-6.35).

## Conclusion

A bundle intervention aimed to prevent CVC-BSI proved to be effective in the ICU setting of an acute-care hospital in the developing world.

## Disclosure of interest

None declared.

